# Dengue-3 Virus Entry into Vero Cells: Role of Clathrin-Mediated Endocytosis in the Outcome of Infection

**DOI:** 10.1371/journal.pone.0140824

**Published:** 2015-10-15

**Authors:** Luana E. Piccini, Viviana Castilla, Elsa B. Damonte

**Affiliations:** 1 Laboratorio de Virología, Departamento de Química Biológica, Facultad de Ciencias Exactas y Naturales, Universidad de Buenos Aires, Buenos Aires, Argentina; 2 IQUIBICEN, Consejo Nacional de Investigaciones Científicas y Técnicas (CONICET), Ciudad Universitaria, Buenos Aires, Argentina; Institut Pasteur of Shanghai, CHINA

## Abstract

The endocytic uptake and intracellular trafficking for penetration of DENV-3 strain H-87 into Vero cells was analyzed by using several biochemical inhibitors and dominant negative mutants of cellular proteins. The results presented show that the infective entry of DENV-3 into Vero cells occurs through a non-classical endocytosis pathway dependent on low pH and dynamin, but non-mediated by clathrin. After uptake, DENV-3 transits through early endosomes to reach Rab 7-regulated late endosomes, and according with the half-time for ammonium chloride resistance viral nucleocapsid is released into the cytosol approximately at 12 min post-infection. Furthermore, the influence of the clathrin pathway in DENV-3 infective entry in other mammalian cell lines of human origin, such as A549, HepG2 and U937 cells, was evaluated demonstrating that variable entry pathways are employed depending on the host cell. Results show for the first time the simultaneous coexistence of infective and non -infective routes for DENV entry into the host cell, depending on the usage of clathrin-mediated endocytosis.

## Introduction

Dengue virus (DENV) is a mosquito-borne member of *Flavivirus*, a genus in the family *Flaviviridae* including important human pathogens like the four DENV serotypes, yellow fever virus (YFV), West Nile virus (WNV), Japanese encephalitis virus (JEV) and tick-borne encephalitis virus (TBEV). DENV-1 to DENV-4 cocirculate in tropical and subtropical regions of America and Asia and can produce a wide spectrum of clinical outcome, from an unapparent infection, the mild and self-limited dengue fever (DF), to the severe forms of dengue hemorrhagic fever and dengue shock syndrome (DHF/DSS).This dengue grave disease is apparently associated to the phenomenon known as antibody-dependent enhancement (ADE) mainly occurring in a secondary infection with an heterologous serotype [1–3]. Due to the high prevalence of dengue illness in the endemic areas, it has turned a serious problem for public health. However, there are no vaccines or antiviral drugs currently available for prevention or treatment of dengue infection. In the past few years, several strategies targeted to different stages of DENV life cycle were intended for antiviral development [4, 5]. In particular, virus entry has become an attractive alternative for therapeutic intervention against viruses, since it represents a barrier to block the beginning of infection and it is determinant of viral tropism and pathogenesis [6–9].

The entry of DENV into the cell appears to be a very complex event regulated by several cell- and virus-dependent factors. In particular, the intracellular route for endocytic uptake and trafficking may be variable according to the type of host cell as well as the virus serotype and/or strain. In the mosquito C6/36 cell line a consistent use of clathrin-mediated endocytosis has been reported for the four DENV serotypes [10, 11], whereas a more variable route of entry may be hijacked by DENV in mammalian cells. In human cells, the internalization of DENV-2, the most extensively studied serotype, was also found to be mediated by the clathrin-dependent endocytic pathway in monocytes [12], HeLa [13], HepG2 [14], ECV304 [15] and A549 [16] cells. By using RNA interference silencing methods, the infection of human hepatic cells Huh7 with the four serotypes DENV-1 to DENV-4 was also shown to be potently inhibited by siRNAs targeting genes associated with clathrin-mediated endocytosis [17]. By contrast, the entry of all DENV serotypes in hepatoma derived HepG2 cells was proposed to occur by multiple pathways, including clathrin endocytosis and macropinocytosis, when a combination of biochemical inhibition and overexpression of dominant negative mutants was employed [18]. With respect to monkey cells, DENV-2 was internalized in BSC-1 cells via clathrin-dependent endocytosis [19], but alternative entry pathways were found in Vero cells: the entry of DENV-2 occurs through a non-classical clathrin- and caveolin-independent process whereas DENV-1 enters to Vero cells via a clathrin-mediated route [16].

This paper presents studies about the endocytic uptake and intracellular trafficking for penetration of DENV-3 into Vero cells. Although DENV-3 reporting has increased rapidly in Asia and America since the 1990s [20], studies on infective entry of this serotype in mammalian cells are very limited: a predominant participation of clathrin-mediated endocytosis was demonstrated for DENV-3 entry only in hepatic cells [17, 18]. Here, the mode of entry of DENV-3 into Vero cells was systematically analyzed by employing several biochemical and molecular inhibitors. The Vero cell line was chosen because it is a host system usually employed to propagate DENV, to evaluate anti-DENV activity of compounds and to produce virus in different strategies for the development of DENV vaccines [21–23]. Furthermore, as above mentioned the possibility of different entry pathways was detected for other DENV serotypes in Vero cells [16]. The results presented show for the first time the possible use of infective and non-infective routes for DENV entry into the host cell, depending on the usage of clathrin-mediated endocytosis. Furthermore, the influence of the clathrin pathway in DENV-3 infective entry in other mammalian cell lines of human origin was also comparatively evaluated demonstrating the ability of this virus to employ variable entry pathways depending on the host cell.

## Materials and Methods

### Cells and Virus

The cell lines Vero (African green monkey kidney, ATCC CCL-81) and A549 (lung carcinoma human cells, ATCC CCL-185) were grown in Eagle's minimum essential medium (MEM) (GIBCO, USA) supplemented with 5% fetal bovine serum. For maintenance medium (MM), the serum concentration was reduced to 1.5%. The C6/36 mosquito cell line from *Aedes albopictus*, adapted to grow at 33°C, was provided by the Instituto Nacional de Enfermedades Virales Humanas (INEVH) Dr. J. Maiztegui (Pergamino, Argentina). This cell line was cultured in L-15 medium (Leibovitz, GIBCO, USA) supplemented with 0.3% tryptose phosphate broth, 0.02% glutamine, 1% MEM non-essential amino acids solution and 5% fetal bovine serum. The human hepatoma cell line HepG2 (ATCC HB-8065) was propagated in MEM containing 0.03% glutamine, 0.01% sodium pyruvate and 10% fetal bovine serum. The human myelomonocytic cell line U937 (ATCC CRL-1593.2) was grown in suspension in RPMI medium 1640 (GIBCO, USA) supplemented with 10% fetal bovine serum.

DENV-3 strain H87, a prototype strain isolated from the Philippines in 1956, was provided by Dr. A.S. Mistchenko, Hospital de Niños Dr. Ricardo Gutiérrez, Buenos Aires, Argentina. The viral stocks were prepared in C6/36 cells and titrated by plaque formation assay in Vero cells.

### Antibodies and Reagents

The anti-DENV antibody was a mouse monoclonal antibody reactive against the E glycoprotein of the four dengue serotypes purchased from Abcam (UK). Goat anti-mouse IgG conjugated to fluorescein isothiocyanate (FITC) or rhodamine (TRITC), and TRITC-cholera toxin B subunit were purchased from Sigma-Aldrich (USA). TRITC- human transferrin was purchased from Molecular probes (USA).

Dansylcadaverine, chlorpromazine, nystatin, methyl-β-cyclodextrin, ammonium chloride, concanamycin A, dynasore, acridine orange and 3-(4,5-dimethylthiazol-2-yl)-2,5-diphenyl tetrazolium bromide (MTT) were purchased from Sigma-Aldrich (USA).

### Drug Inhibitory Treatments

The non-cytotoxic treatment conditions with each pharmacological inhibitor were first determined. Cells were treated with different compound concentrations during 3 h and thereafter cell viability was measured by MTT assay, as described previously [24]. Cell monolayers were pretreated for 1 h at 37°C with the non-cytotoxic concentrations of each inhibitor in MM or in MEM without serum for the cholesterol-reactive compounds nystatin and methyl-β-cyclodextrin. Then, cells were infected at an m.o.i. of 1 PFU/cell in the presence of the drug, except for nystatin and methyl-β-cyclodextrin where drug-pretreated cultures were extensively washed before infection to eliminate compound and were further incubated in the absence of compound. After 1 h at 37°C virus inocula were removed, cells were washed with PBS and the number of internalized virions was determined by an infectious centre assay. Briefly, cell monolayers were treated with proteinase K (1 mg/ml, Invitrogen) for 45 min at 4°C to remove adsorbed but not internalized virus. Then, proteinase K was inactivated with 2 mM phenyl-methyl-sulfonyl fluoride in PBS with 3% bovine seroalbumin (BSA), cells were washed with PBS 0.2% BSA by low speed centrifugation, and cell pellets were resuspended in MM. Different serial dilutions of the cell suspensions were plated onto Vero cell monolayers to quantify internalized virus by plaque formation at 7 days p.i..

To determine the effect of pharmacological inhibitors on virus yield or viral antigen expression, cells were pretreated with each compound, and then infected with DENV-3 in the presence of the drug. In all cases, virus inocula were removed after 1h at 37°C, then cultures were extensively washed with PBS and further incubated at 37°C for 48 h in MM without inhibitor. These experimental conditions assessed that the effect of the compound was exerted only during the initial entry process in the first hour of infection. Then, supernatants were collected to evaluate extracellular virus yields by plaque formation and cells were processed for immunofluorescence assay as described below.

To determine the number of internalized viral RNA molecules, infected cells were processed as above mentioned for the internalization assay and total RNA was extracted from cells by using TRIzol (Invitrogen, USA) according to the manufacturer’s instructions. The amount of internalized viral RNA was quantified by a real time RT-PCR assay utilizing Taq Man technology as previously described [24].

To assess the efficiency of blockade of each endocytic pathway by the different inhibitors, uptake studies with TRITC-transferrin (chlorpromazine, dansylcadaverine, dynasore) or FITC-cholera toxin B subunit (nystatin, methyl-β-cyclodextrin) and acridine orange (ammonium chloride, concanamycin A) were performed. Cells treated or not with inhibitors were incubated with 15 μg/ml TRITC-transferrin, 0.3 μg/ml FITC-cholera toxin or 1 μg/ml acridine orange during 1 h at 37°C. Then, cells were processed for visualization in a fluorescence microscope as described below.

### Penetration Kinetics by Resistance to Ammonium Chloride Treatment

Vero cells were infected with 100–200 PFU/well of DENV-3 and virus binding was synchronized at 4°C for 1 h. Then, cells were washed with cold PBS and rapidly shifted at 37°C by addition of pre-warmed MM to initiate virus internalization. Ammonium chloride 50 mM was added at indicated times and kept throughout the infection. After 3 h of the temperature shift, cells were treated with citrate buffer (40 mM citric acid, 10 mM KCl, 135 mM Na Cl, pH 3) for 1 min to inactivate adsorbed but not internalized virus. It was previously corroborated that treatment of a suspension of DENV-3 virions with citrate buffer during 1 min produced 99.8% inactivation of infectivity, determined by plaque formation assay. Finally, cells were washed with PBS and covered with plaquing medium. Plaques were counted at 7 days p.i..

### Transfection of Dominant Negative (DN) Mutants

The GFP tagged constructs of Eps15 GFP-EH29 (DN mutant) and GFP-DIIIΔ2 (control) were kindly provided by Dr. C. Shayo (IBYME, Argentina). The GFP tagged constructs of wild-type caveolin-1 GFP-cav-1 wt and DN mutants GFP-cav-1 DN and GFP-cav-1 Y14F were kindly provided by Dr. J. M. Bergelson (University of Pennsylvania, USA). GFP-dyn II wt and DN mutant GFP-dynII-K44A were kindly provided by Dr. M. A. McNiven (Mayo Clinic, USA). The GFP tagged constructs of Rab5 and Rab7 wt and DN mutants S35N and T22N, respectively, were kindly provided by Dr. M. I. Colombo (Universidad Nacional de Cuyo, Argentina). Vero cells grown on cover slips until 70% confluency were transfected with 4 μg of each construct using Lipofectamine 2000 (Invitrogen, USA) in Opti-MEM. The DNA-liposome complexes were added to the cells, cultures were incubated for 6 h at 37°C, and then medium was replaced by MM. At 24 h post-transfection, cell cultures were infected with DENV-3 (m.o.i of 1 PFU/cell), and after 24 h infection cultures were fixed with methanol for 10 min at -20°C and stained for immunofluorescence as described below.

### Immunofluorescence Assay

After methanol fixation, cells were washed with PBS and stained for DENV with a monoclonal antibody against E glycoprotein (1:300 dilution) for 30 min at 7°C. Then, cells were washed with PBS and incubated with TRITC-labelled goat anti-mouse IgG (1: 300 dilution) in plasmid-transfected cultures or FITC-labelled goat anti-mouse IgG (1: 100 dilution) in drug treated cultures for 30 min at 37°C. After a final washing with PBS, cells were mounted in a glycerol solution containing 1,4-diazabicyclo[2,2,2]octane and visualized under a fluorescence microscope Olympus BX51.

In transfected cultures, the percentage of infection of transgene-expressing cells was calculated by scoring the number of cells positive for viral antigen from 250 transfected cells with comparable levels of transgene expression. In infected cells treated with the pharmacological inhibitors, the percentage of fluorescent cells in each sample was calculated from 25 microscope fields selected at random.

### Statistical Analyses

Statistical analyses were performed using GraphPad Prism software. Comparison of means was tested by Student´s unpaired t-test. Statistical significance is depicted in figures: * *p* < 0.05, ** *p* < 0.01, *** *p* < 0.001.

## Results

### Clathrin is not Required for Infective DENV-3 Entry into Vero Cells

**Fig 1:**
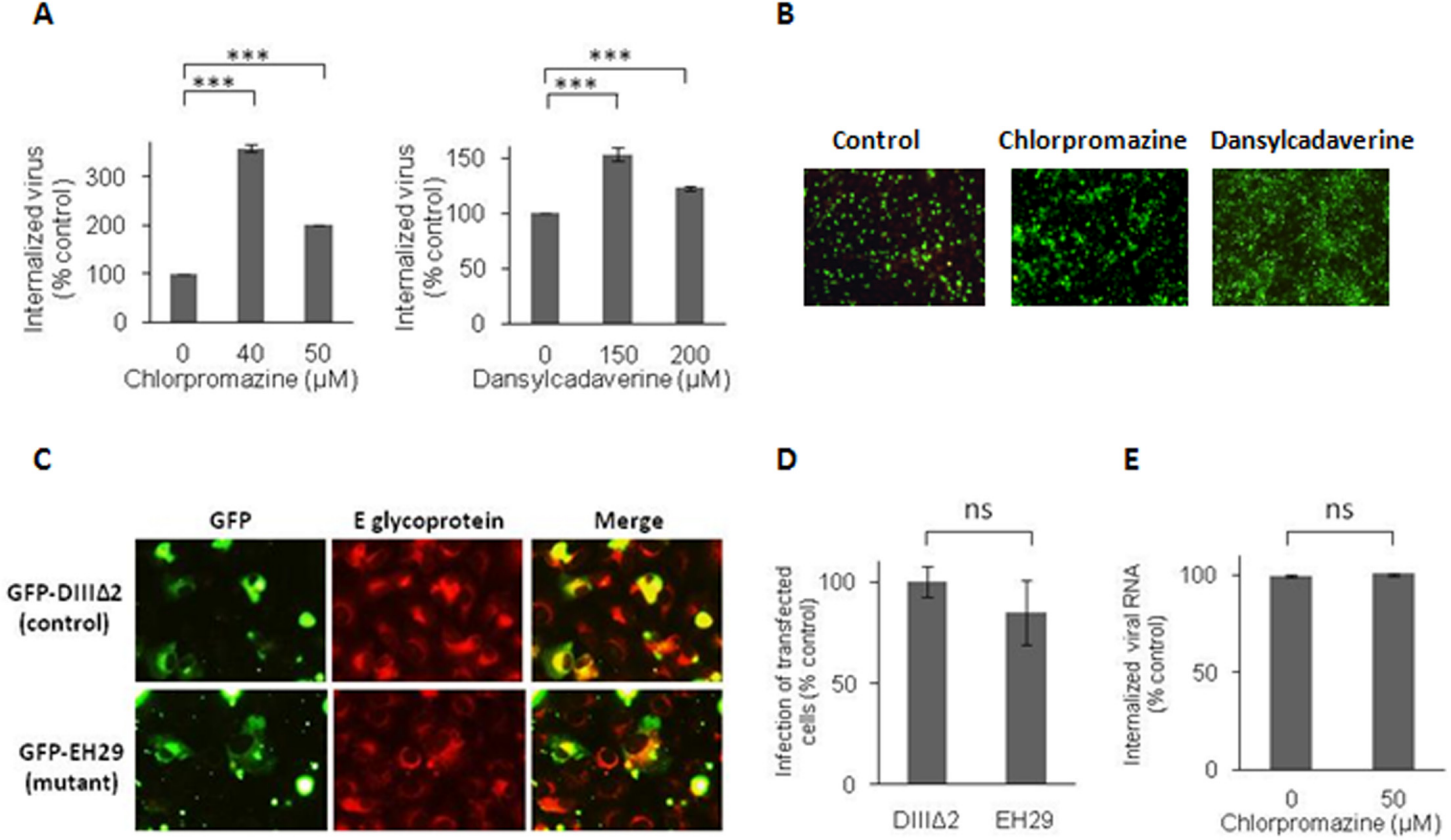
Clathrin is not required for DENV-3 infective internalization into Vero cells. (A) Cells were treated with chlorpromazine or dansylcadaverine and infected with DENV-3. After 1h of internalization in presence of the drugs, monolayers were treated with proteinase K and the cell pellets were plated onto Vero cells to determine internalized virus by an infectious centre assay. (B) Cells treated with 40 μM chlorpromazine, 150 μM dansylcadaverine or untreated (control) were infected with DENV-3. At 48 h p.i., immunofluorescence staining was carried out using mouse anti-E glycoprotein antibody. (C) Cells transiently transfected with GFP-DIII∆2 or GFP-EH29 were infected with DENV-3. After 24 h cells were fixed and viral antigen expression was visualized by immunofluorescence staining using mouse anti-E glycoprotein antibody and TRITC-labelled anti-mouse IgG. (D) For quantification of samples shown in C, 250 transfected cells with similar levels of GFP expression were screened and cells positive for viral antigen were scored. (E) Vero cells were infected and processed as in (A) to obtain cell pellets, then total RNA was extracted and real-time RT-PCR was performed to determine the amount of internalized viral RNA molecules. In (A), (D) and (E) results are expressed as the mean of three independent experiments ± SD. Asterisks indicate statistical significance (*** *p* < 0.001), ns: non-significant difference between treated sample and control.

The clathrin-mediated route of endocytosis is the main cellular pathway employed by viruses for cell entry and it has been reported as a differential host factor for DENV-1 and DENV-2 entry [16]. Thus, our initial experimental approach was to analyze whether DENV-3 enters Vero cells through this pathway by determining the effects on virus internalization of chlorpromazine, a very well-known cationic amphiphilic drug affecting the assembly of clathrin lattices on cell surface and on endosomes [25]. No reduction in the amount of internalized virions after 1 h of infection at 37°C in the presence of non-cytotoxic concentrations of chlorpromazine was detected by the proteinase K-resistant infectious center assay (Fig 1A). In fact, a significant enhancement was observed in infectious DENV-3 particles inside the cell when the clathrin-pathway was blocked, with an increase in percentage of internalized virus up to 300%. The lack of clathrin-dependence was further confirmed with dansylcadaverine, other pharmacological inhibitor used for blockade of clathrin-dependent endocytosis by affecting the clustering of membrane-bound ligands or particles in clathrin coated pits [26–29]. The effect of dansylcadaverine on DENV-3 infective internalization in Vero cells was similar to that observed with chlorpromazine (Fig 1A).

The action of both drugs on clathrin-mediated endocytosis was effectively assessed by determining the blockade in the internalization of TRITC-labelled transferrin, a typical ligand known to enter into the cell by this endocytic route, after Vero cell treatment with both compounds. The fluorescence pattern inside the cell cytoplasm observed in control untreated cultures was completely lost when cells were treated with chlorpromazine or dansylcadaverine (S1 Fig).

The effect of these two clathrin inhibitors on DENV-3 infection was also assayed on the expression of viral E glycoprotein by indirect immunofluorescence. Cells were treated with compounds only during the first hour of infection and then washed and incubated for 48 h p.i. in the absence of inhibitor. When cells were treated with chlorpromazine or dansylcadaverine during virus entry, no inhibition in viral antigen-positive cells was observed. By contrast and accordingly with infective internalization data, a significant increase of 316.4% and 147.8% was observed in the number of cells expressing E glycoprotein in 40 μM chlorpromazine- and 150 μM dansylcadaverine-treated cultures, respectively in comparison to untreated infected cells.

Next, we used wt and DN mutant constructs of the clathrin-coat associated cellular protein Eps15 as specific molecular tools to confirm the lack of participation of the clathrin pathway in the infective entry of DENV-3. Overexpression of the DN mutant of Eps15 selectively interferes with clathrin-coated pit assembly at the plasma membrane without affecting other endocytic pathways [30]. Vero cells were transfected with plasmids expressing GFP-tagged wt Eps15 (GFP-DIIIΔ2) or GFP-tagged DN Eps15 (GFP-EH29). When transfected cells were infected with DENV-3, it can be observed that the presence of the mutant protein did not affect infection since similar signals for merge images of GFP and E glycoprotein were seen in DN and wt transfected cells at 48 h p.i. (Fig 1C). The percentage of infection was similar in cultures transfected with wt or DN Eps15 (Fig 1D). As with the pharmacological inhibitors, the functionality of the molecular constructs was confirmed by incubation of transfected cells with TRITC-labeled transferrin. The transferrin uptake was inhibited by the expression of the DN Eps15 protein (S1 Fig).

A significant increase in the number of internalized virions was observed when clathrin-mediated endocytosis was blocked (Fig 1A and 1B). Trying to understand the reasons for this improvement in viral entry, we determined, in parallel, by quantitative RT-PCR the amount of viral RNA molecules internalized in presence or absence of chlorpromazine, in the same conditions of the infectivity assay presented in Fig 1A. As seen in Fig 1E, similar amounts of viral RNA were taken by the cell with or without clathrin inhibition.

### The Endocytic Entry of DENV-3 to Vero Cells Depends on Dynamin

**Fig 2:**
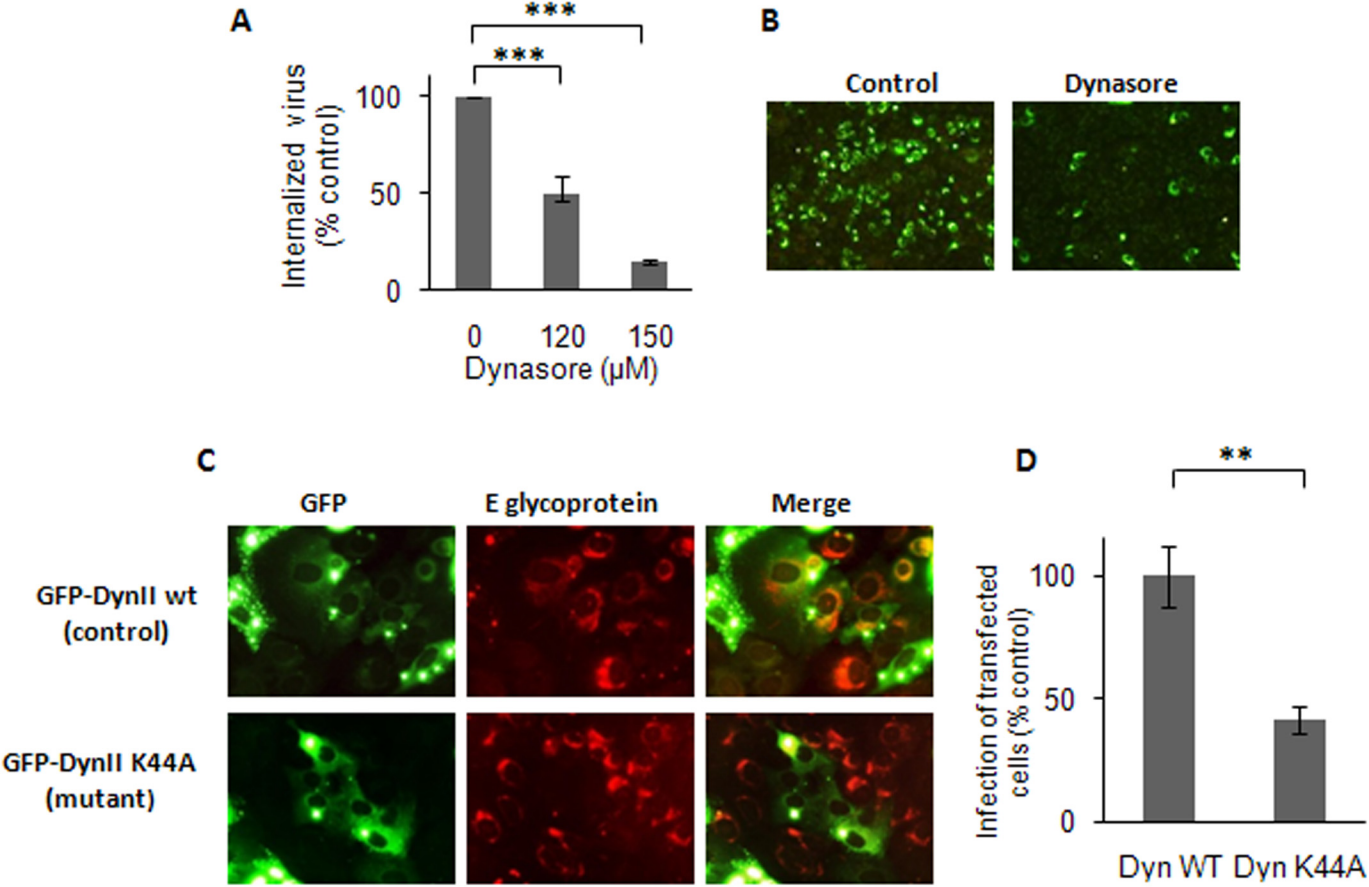
DENV-3 entry into Vero cells is dependent on dynamin. (A) Cells were treated with dynasore and infected with DENV-3. After 1h of internalization in presence of the drug, monolayers were treated with proteinase K and the cell pellets were plated onto Vero cells to determine internalized virus by an infectious centre assay. (B) Cells treated with 150 μM dynasore or untreated (control) were infected with DENV-3. At 48 h p.i., immunofluorescence staining was carried out using mouse anti-E glycoprotein antibody. (C) Cells transiently transfected with GFP-Dyn II wt or GFP-Dyn II K44A were infected with DENV-3. After 24 h, cells were fixed and viral antigen expression was visualized by immunofluorescence staining using mouse anti-E glycoprotein antibody and TRITC-labelled anti-mouse IgG. (D) For quantification of samples shown in C, 250 transfected cells with similar levels of GFP expression were screened and cells positive for viral antigen were scored. In (A) and (D) results are expressed as the mean of three independent experiments ± SD. Asterisks indicate statistical significance (** *p* < 0.01, *** *p* < 0.001).

Dynamin is a cellular GTPase essential for scission of endocytic vesicles from the plasma membrane and it is usually required for classical clathrin-dependent endocytosis and for some non-classical pathways exploited by viruses [31]. The participation of dynamin in DENV-3 entry into Vero cells was analyzed by evaluating the effect of dynasore, an inhibitor of dynamin GTPase activity [32]. A dose-dependent and strong inhibition of DENV-3 internalization was detected in Vero cells treated with dynasore before and during the first hour of virus infection (Fig 2A). As control of dynasore specific effect, the uptake of transferrin was also blocked in the presence of the drug (S2 Fig). Consistent with the results obtained about DENV-3 internalization, virus protein expression determined by immunostaining at 48 h p.i. was also inhibited by dynasore treatment only during the first hour of infection when virus entry takes place (Fig 2B). A reduction of 95.4% was detected in the number of cells expressing DENV-3 E protein in cultures treated with dynasore (150 μM) in comparison to untreated infected ones.

Finally, the participation of dynamin in DENV-3 entry into Vero cells was corroborated by using the GFP-tagged versions of the wt form of dynamin II GFP-dyn II and the DN mutant GFP-dyn II K44A [33, 34]. Vero cells were transfected with both constructs and after 24 h of transfection cells were either incubated with TRITC-transferrin, as positive control of the expression of wt and DN constructs, or infected with DENV-3. The expression of the dynamin mutant K44A produced a high inhibition in the uptake of transferrin (S2 Fig), as well as a significant reduction in the number of transfected infected cells relative to the control protein expression (Fig 2C and 2D).

### Participation of Caveola-mediated Pathway on DENV-3 Entry

**Fig 3:**
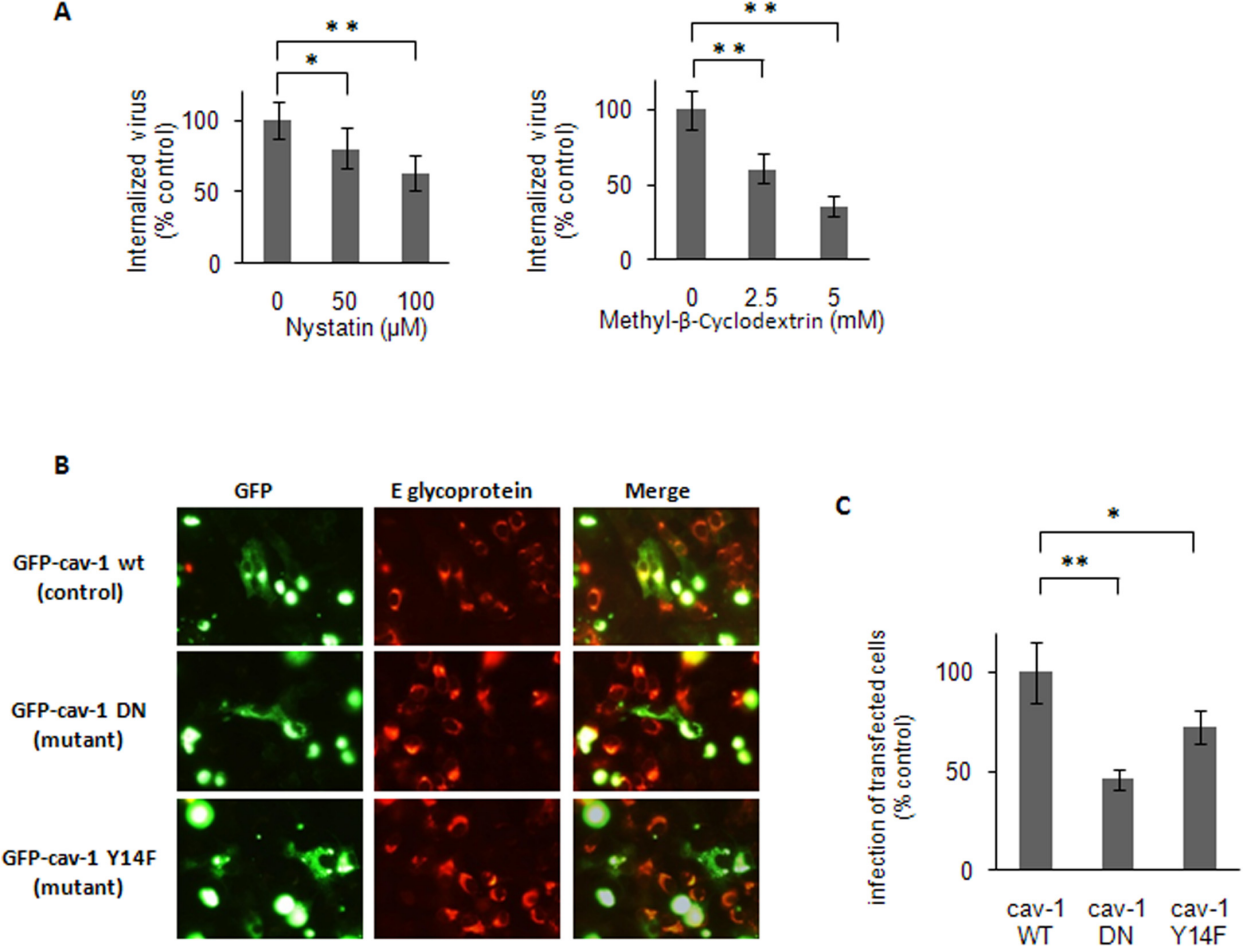
Caveola-dependence for DENV-3 entry. (A) Cells were pretreated with nystatin or methyl-β-cyclodextrin. Then monolayers were washed and infected with DENV-3. After 1h of internalization, cells were treated with proteinase K and the cell pellets were plated onto Vero cells to determine internalized virus by an infectious centre assay. (B) Cells transiently transfected with GFP-cav-1 wt, GFP-cav-1 DN or GFP-cav-1 Y14F were infected with DENV-3. After 24 h, cells were fixed and viral antigen expression was visualized by immunofluorescence staining using mouse anti-E glycoprotein antibody and TRITC-labelled anti-mouse IgG. (C) For quantification of samples shown in B, 250 transfected cells with similar levels of GFP expression were screened and cells positive for viral antigen were scored. In (A) and (C) results are expressed as the mean of three independent experiments ± SD. Asterisks indicate statistical significance (* *p* < 0.05, ** *p* < 0.01).

The main clathrin-independent endocytic route used by virus is the uptake mediated by caveolae, vesicles arising from specialized lipid rafts [35]. Membrane cholesterol is a prominent component of lipid rafts and, consequently, cholesterol-interacting drugs that disrupt the lipid bilayer impair caveola-dependent endocytosis. The effects on DENV-3 infection of depletion of cholesterol by methyl-β-cyclodextrin [36, 37] or fixation of cholesterol by complex formation with nystatin [38] was next analyzed. As it is known that both inhibitors are strong inactivating agents of DENV serotypes infectivity, including DENV-3 [39], cells were pretreated with the compounds before virus infection to avoid a direct contact between drug and virions. These treatment conditions effectively altered lipid raft/caveolae organization since the uptake of TRITC-labelled cholera toxin, a marker of internalization through this route, was affected (S3 Fig). A significant but not total reduction in DENV-3 internalization was detected in Vero cells pretreated with methyl-β-cyclodextrin or nystatin, reaching about 40–60% inhibition at the highest concentrations tested (Fig 3A).

The influence of the expression of GFP-tagged DN mutants for caveolin-1, the main structural protein in caveolae, on DENV-3 infection was also evaluated. Two DN mutants for caveolin-1 were employed. The mutant designated GFP-cav-1 DN presents the fusion of GFP at the N-terminal domain of caveolin-1, a situation that inhibits the functionality of the protein, whereas the GFP fusion at the C-terminal domain, designated GFP-cav-1 wt, does not affect the protein [40]. Other DN mutant named GFP-cav-1 Y14F was obtained by a point mutation that prevents caveolin-1 phosphorylation [41]. The transfection of Vero cells with both mutants GFP-cav-1 DN and GFP-cav-1Y14F produced a significant reduction in the percentage of DENV-3 infected transfected cells in comparison to wt-transfected ones (Fig 3B and 3C).

### Dependence of DENV-3 Entry on Acid pH and Site for Virion Uncoating

**Fig 4:**
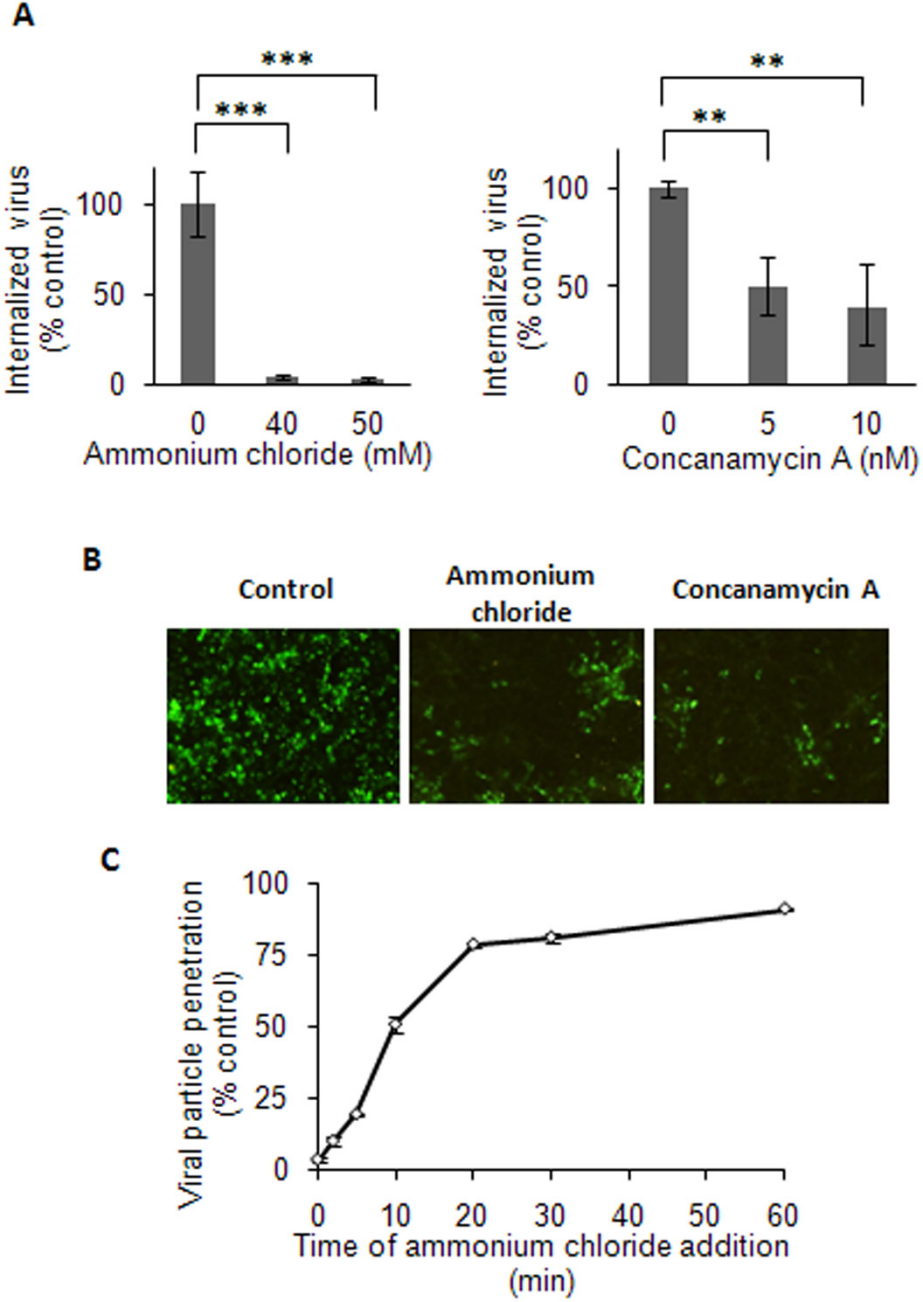
Low pH-dependence of DENV-3 entry into Vero cells. (A) Cells were treated with ammonium chloride or concanamycin A and infected with DENV-3. After 1h of internalization in presence of the drugs, monolayers were treated with proteinase K and the cell pellets were plated onto Vero cells to determine internalized virus by an infectious centre assay. (B) Cells treated with 50 mM ammonium chloride, 10 nM concanamycin A or untreated (control) were infected with DENV-3. At 48 h p.i., immunofluorescence staining was carried out using mouse anti-E glycoprotein antibody. (C) 100–200 PFU/well of DENV-3 were bound to cells at 4°C and then allowed to internalize by warming at 37°C. Ammonium chloride was added at different times post-temperature shift. After 3h of incubation at 37°C, extracellular virus was inactivated with citrate buffer and cells were overlaid with plaquing medium. Plaque number was normalized to the values in control cultures without ammonium chloride. In (A) and (C) results are expressed as the mean of three independent experiments ± SD. Asterisks indicate statistical significance (** *p* < 0.01, *** *p* < 0.001).

Most enveloped viruses entering by endocytosis require a defined low pH vesicular environment to trigger fusion of the viral envelope with the endosomal membrane and deliver the nucleocapsid into the cytosol. To test the requirement of low pH for DENV-3 entry to Vero cells, the effect of two compounds was first determined: ammonium chloride, a weak base that immediately raises the pH of intracellular acidic vesicles [42], and concanamycin A, an inhibitor of the vacuolar proton ATPase [43]. Pretreatment of Vero cells either with ammonium chloride or with concanamycin A produced a very important reduction of DENV-3 internalization (Fig 4A) as well as a consequent inhibition of viral antigen expression (Fig 4B). The effect of both compounds on intracellular acidic vesicles was assured by treatment of uninfected Vero cells in the same conditions used for study of virus internalization and subsequent staining with acridine orange. Under a fluorescence microscope the rise in vesicular pH was observed (S4 Fig).

To better precise the timing of the acid-requiring step during endocytic pathway we performed a kinetics assay of resistance to ammonium chloride [44, 45], as described in Materials and Methods. As shown in Fig 4C, infective virion penetration started at 5 min post-incubation at 37°C and reached a half-maximal level within 12 min. This half time for ammonium chloride resistance, equivalent to viral nucleocapsid escape from endosomes, resembles that of the late penetrating viruses`[16, 19, 31, 44], suggesting a membrane fusion in late endosomes for DENV-3.

**Fig 5:**
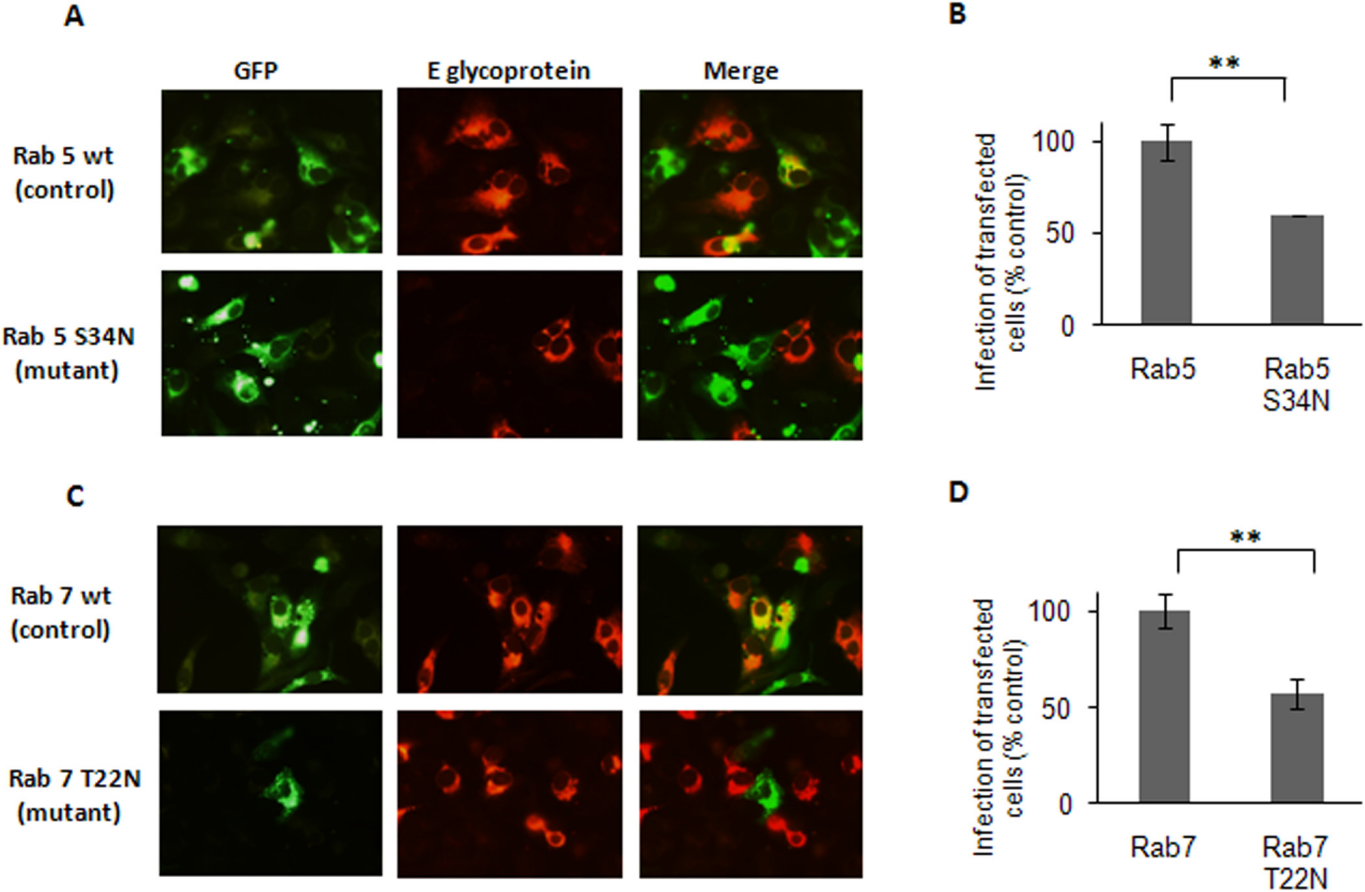
Transport of DENV-3 particles to early and late endosomes. Cells transiently transfected with the GFP-tagged versions of Rab5 wt and S34N (A, B) or Rab7 wt and T22N (C, D) were infected with DENV-3. After 24 h cells were fixed and viral antigen expression was visualized by immunofluorescence staining using mouse anti-E glycoprotein antibody and TRITC-labelled anti-mouse IgG. (B)(D)For quantification of samples, 250 transfected cells with similar levels of GFP expression were screened and cells positive for viral antigen were scored. Results are expressed as the mean of three independent experiments ± SD. Asterisks indicate statistical significance (** *p* < 0.01).

To further analyze the endocytic trafficking of DENV-3 in Vero cells and identify the probable cellular vesicle where the fusion event for DENV-3 penetration may take place, the role of different Rab GTPases regulators of transport to endocytic vesicles was evaluated. To this end, cells were transfected with plasmids expressing GFP-tagged versions of wt and DN S34N forms of Rab5 and wt and DN T22N forms of Rab7. Both Rab5 and Rab7 GTPases are known to be involved in vesicular trafficking to early and late endosomes, respectively [46, 47]. At 24 h post-transfection cells were infected with DENV-3, and 24 h later, GFP and viral antigen expression were detected. In wt-transfected cells, superposition of GFP and DENV-3 protein fluorescence was observed whereas the overexpression of both DN mutants Rab5 S34N and Rab7 T22N significantly reduced the number of infected transfected cells (Fig 5A–5D), indicating the involvement of trafficking to early and late endosomes for successful DENV-3 infection.

### Clathrin-dependence for DENV-3 Entry is Dependent on the Host Cell

By contrast to the mode of DENV-3 entry into Vero cells here shown characterized by a significant increase in infective virus internalization when the clathrin-mediated pathway is blocked, our previous studies reported that infectious DENV-3 entry into mosquito C6/36 cells, similarly to DENV-1, DENV-2 and DENV-4 serotypes, occurred by clathrin-mediated endocytosis [11]. So, we decided to extend the evaluation of clathrin-dependence for DENV-3 entry to other mammalian cells in comparison to Vero cells. We tested the effect of chlorpromazine as marker of entry through clathrin-pathway because, according to results shown in Fig 1 and S1 Fig, it affected specifically clathrin-mediated endocytosis and the results obtained with this drug were corroborated with other pharmacological and molecular inhibitors. The hepatoma HepG2, the lung carcinoma A549 and the myelomonocytic U937 human cell lines were tested. First, the range of cytotoxicity and effectiveness of chlorpromazine to block transferrin uptake was determined. The cytotoxicity of chlorpromazine for A549 cells was similar to that observed in Vero cells whereas HepG2 and U937 cells were more susceptible and the maximum non-cytotoxic concentration in both cell lines was 20 μg/ml. Chlorpromazine affected transferrin uptake in the three cell systems as effectively as in Vero cells (S1 Fig). Then, cultures of the three cell lines, in parallel to Vero cells, were treated or not with non-cytotoxic concentrations of chlorpromazine before infection and during the first hour of infection, and at 48 h p.i. virus yields were titrated.

**Fig 6:**
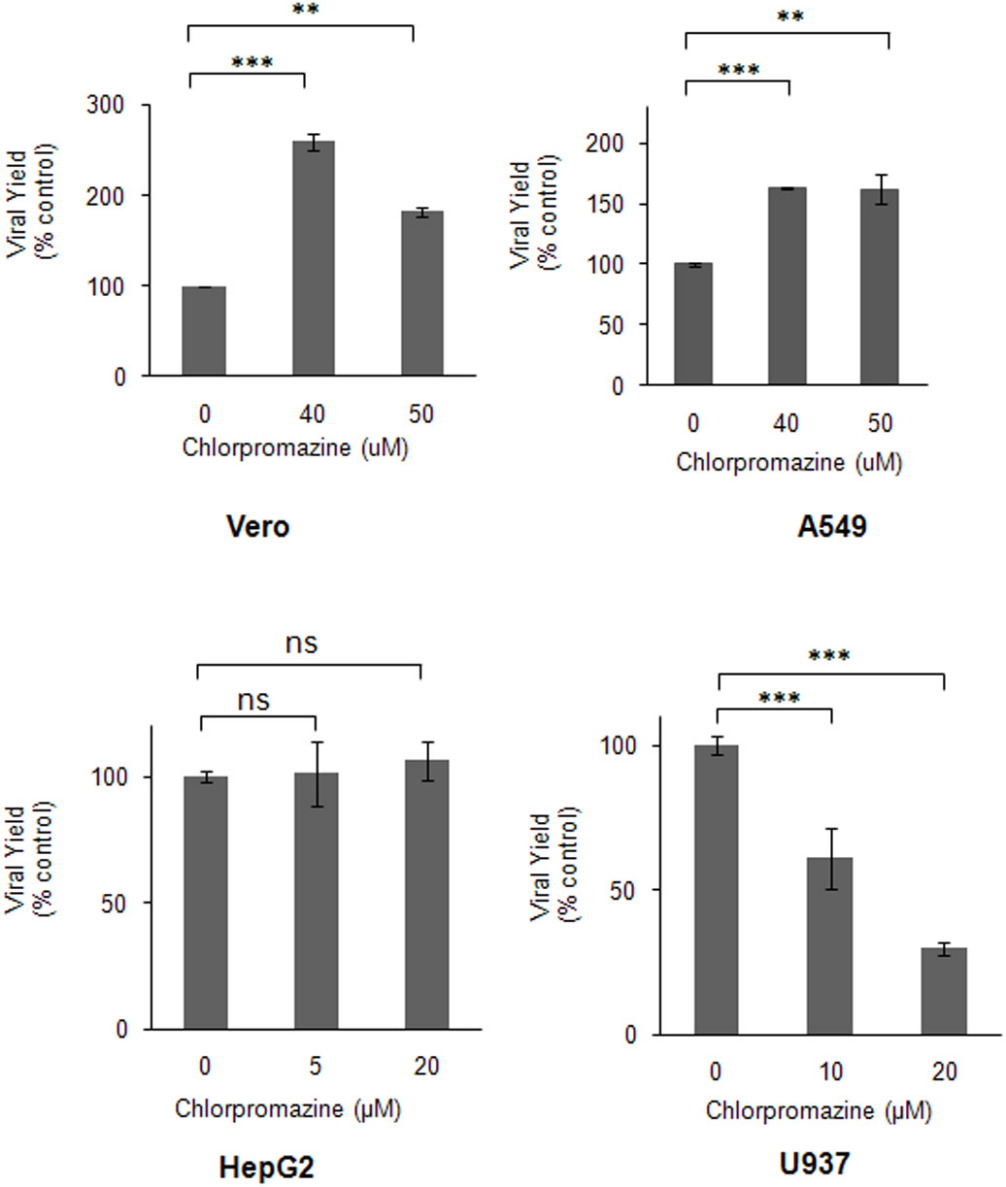
Effect of chlorpromazine on DENV-3 infection of different host cells. Vero, A549, HepG2 and U937 cells were treated with chlorpromazine and infected with DENV-3. At 48 h p.i., virus yields were determined by plaque formation in Vero cells. Results are expressed as the mean of three independent experiments ± SD. Asterisks indicate statistical significance (** *p* < 0.01, *** *p* < 0.001). ns: non-significant difference between treated sample and control.

The results were variable, according to the cell system (Fig 6). A very important increase in virus production was detected in Vero and A549 cells by inhibition of the clathrin route; in hepatic HepG2 cells virus yields were unaffected by chlorpromazine treatment whereas in the U937 cell line there was a strong dose-dependent inhibition, suggesting a clathrin-mediated endocytosis.

## Discussion

Most enveloped viruses utilize an endocytic mechanism to gain entry into a permissive cell and start infection. The results presented in this paper show that the infective entry of DENV-3 into Vero cells occurs through a non-classical endocytosis pathway dependent on low pH- and dynamin but non-mediated by clathrin. After uptake, DENV-3 transits through early endosomes to reach Rab 7-regulated late endosomes and viral nucelocapsid is released into the cytosol approximately at 12 min post-infection.

The main finding of our study is the demonstration of the involvement of clathrin in the endocytic pathway for DENV-3 entry into Vero cells as a key point for the outcome of infection. The comparison of the amounts of infective internalized virus, determined by an infectious centre assay, and the viral RNA molecules, measured by quantitative RT-PCR, in the presence of biochemical inhibitors of the clathrin-mediated endocytosis shows a significant and outstanding difference: the infective internalized virions are 200–300% increased whereas the number of viral RNA molecules remained unaffected (Fig 1). Consequently, it appears that DENV-3 can enter into Vero cells by two alternative routes considering clathrin-dependence, but only the non-clathrin-dependent via is an infective route of entry leading to productive viral infection. Thus, when the clathrin-mediated pathway is blocked there is an enhancement in the utilization by DENV-3 of the non-clathrin route resulting in higher efficiency of infection. Interestingly, clathrin-independent endocytosis is a field of investigation that has gained interest in the last decade, from considering it non-existing until its acceptance as a process with a high degree of complexity in mechanisms and regulations [48, 49].

This dual possibility of DENV-3 entry mechanism is not exclusive for Vero cells because a similar increase in virus yield was observed in human A549 cells in the presence of chlorpromazine (Fig 6). By contrast, a different role for clathrin-endocytosis in DENV-3 entry was detected in other human cells. Clathrin-mediated uptake appears not to be involved in any way for DENV-3 entry into HepG2 cells whereas the clathrin-dependent pathway is the infective route of DENV-3 entry in the monocytic U937 cells given the potent inhibitory effect of chlorpromazine treatment. Altogether our data indicate that diverse entry modes can be exploited by DENV-3 depending on the cell type. At present, the molecular basis for the variations in clathrin utilization for DENV-3 entry into diverse host cells cannot be fully elucidated and further studies are required to characterize the cellular components involved in the endocytic pathway observed in different cells. A possible explanation may be proposed in terms of the usage of different virus receptors, since this initial interaction at the cell surface is determinant of the subsequent mechanisms of uptake, trafficking and uncoating of virion inside the cell. However, it is not easy to prove this proposal since the precise nature of the cell receptor/s is still unclear and diverse molecules, including proteins, carbohydrates and lipids, have been reported as putative DENV receptors in the last decades in different host cells, including those cell lines here evaluated for DENV-3 internalization [50].

Likewise, the existence of alternative pathways for internalization has been reported for other human viruses. Among flaviviruses, several studies have demonstrated clathrin-mediated entry for uptake of the serotype DENV-2 in diverse types of mammalian cells [50], but in Vero cells DENV-2 is able to utilize a non-classical internalization route independent of clathrin with subtle differences in comparison to DENV-3 performance here shown [16]. Similarly, Japanese encephalitis virus (JEV) infects fibroblasts in a clathrin-dependent manner, but it deploys a clathrin-independent mechanism to infect neuronal cells [28, 51]. Furthermore, diverse RNA and DNA viruses from other families such as reovirus [52], human immunodeficiency virus [53], equine herpes virus [54], human papillomavirus [55] and herpes simplex virus [56] are able to employ multiple entry modes depending on the type of host cell. Other viruses also can enter in the same host cell by more than one endocytic pathway, one of them is predominant over the other, but both routes guide to a productive infection. This is the case for influenza virus: clathrin-endocytosis and macropynocitosis can operate simultaneously in HeLa, A549 and other non-human cells, with a predominance apparently regulated by the presence of serum, and the specific down-regulation of the clathrin pathway does not affect the total number of entry events [57]. Similarly, it was recently demonstrated that the entry of Ebola virus particles into HeLa cells mainly followed the characteristics of macropinocytosis but, in addition, a smaller fraction of particles entered via clathrin-mediated endocytosis [58]. However, in the mentioned virus-cell examples, the concurrent entry routes are redundant and independent. By contrast, the present findings provide the first evidence for a non-infective uptake of a flavivirus in mammalian cells operating simultaneously and interfering with the productive via of penetration. Probably, the virus has evolved to exploit different cellular pathways in order to increase the host range, but in some cells, like Vero and A549 in this case for DENV-3, this wide usage of internalization options is a disadvantage due to a blockade for complete virus cycle progression.

Our studies have also shown the participation of acid pH, dynamin and caveolae for DENV-3 entry into Vero cells. With respect to caveolae, the inhibition observed by treatment with cholesterol-reactive drugs like nystatin and methyl-β-cyclodextrin or overexpression of DN mutants of caveolin-1 was incomplete (Fig 3). Then, caveolar pathway appears to be a secondary or alternative route of infective DENV-3 uptake that is more predominantly dependent on acid pH and dynamin. In the same manner, a recent study has demonstrated that JEV, another member of *Flaviridae*, also enters into neuroblastoma cells via a clathrin-independent, pH-dependent, dynamin- and caveola-mediated endocytosis [28].

After cellular uptake, enveloped viruses transit through the vesicular transport system to reach the cell vesicle wherein virion envelope uncoating takes place. After evaluation of the requirement of Rab5 and Rab7 GTPase expression with DN mutants, our data allow to conclude that, after transit by early endosomes, DENV-3 particles are sorted to Rab7-dependent late endosomes to productively infect Vero cells (Fig 5), instead of leaving the endosomal pathway immediately after early endosome formation. Accordingly with this location of virus in late endosomes, the kinetics of infectivity resistance to ammonium chloride proved that DENV-3 virion uncoating occurs approximately at 12 min post-infection, a time of endosome escape corresponding to a late virus penetration [59]. For other flaviviruses, contrasting data have been reported about fusion and release of viral nucleocapsid at early [13, 49] or late endosomes [10, 17, 19, 60], apparently also dependent on the virus serotype/strain and the host cell. The present study about the probable DENV-3 penetration from late endosomes is on line with the unique lipid composition, enriched in anionic lipids, reported as a requisite to assure DENV-2 fusion [61].

The results presented in this paper confirm previous studies about the complexity of the process of DENV entry into the host cell, apparently controlled by various cell- and virus-dependent factors and characterized by multiple alternative routes for internalization and trafficking inside the cell until virion uncoating is attained. A new perspective is here provided by the possible simultaneous use of productive and non-productive routes for DENV-3 entry into a cell with a central participation of clathrin-mediated endocytosis to regulate the efficiency of infection, since the delivery of the virion to the final acidic compartment via one endocytic pathway or another may be determinant for infectivity and virus production, at least in Vero and A549 cells.

## Supporting Information

S1 Fig Control of inhibition of clathrin-mediated endocytosis. A. Vero cells were treated with 40 μM chlorpromazine, 150 μM dansylcadaverine or untreated (control) and incubated with TRITC-labelled transferrin. B. Vero cells transiently transfected with GFP-DIII∆2 or GFP-EH29 were incubated with TRITC-labelled transferrin. C. Cells were treated with 40 μM (A549) or 20 μM (HepG2 and U937) chlorpromazine and incubated with TRITC-labelled transferrin.
(TIF)

S2 Fig Control of dynamin inhibition. A. Vero cells were treated with 150 μM dynasore or untreated (control) and incubated with TRITC-labelled transferrin. B. Vero cells transiently transfected with GFP-Dyn II wt or GFP-Dyn II K44A were incubated with TRITC-labelled transferrin.
(TIF)

S3 Fig Control of inhibition of caveola-mediated endocytosis. A. Vero cells were treated with 100 μM nystatin, 5 mM methyl-β-cyclodextrin or untreated (control) and incubated with TRITC-labelled cholera toxin subunit B. B. Vero cells transiently transfected with GFP-cav-1 wt, GFP-cav-1 DN or GFP-cav-1 Y14F were incubated with TRITC-Labelled cholera toxin subunit B.
(TIF)

S4 Fig Control of inhibition of intracellular vesicle acidification. Vero cells were treated with 50 mM ammonium chloride, 10 nM concanamycin A or untreated (control) and stained with acridine orange.
(TIF)
