# Supplementary figures and images for: Dengue-3 Virus Entry into Vero Cells: Role of Clathrin-Mediated Endocytosis in the Outcome of Infection
Source: PLoS One. 2015 Oct 15;10(10):e0140824. doi: 10.1371/journal.pone.0140824 (PMC4607419; doi:10.1371/journal.pone.0140824)

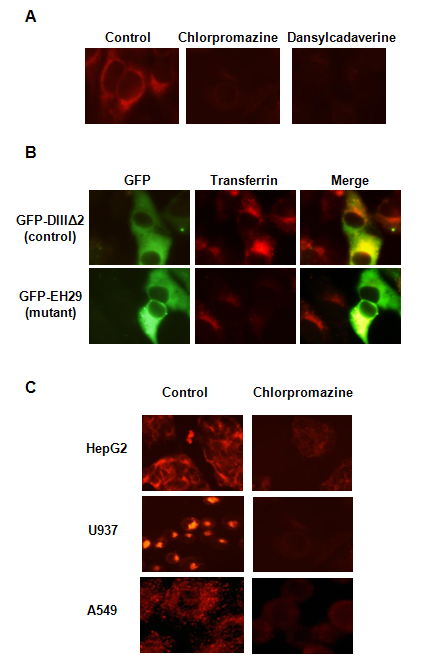

Supplement: S1 Fig — A. Vero cells were treated with 40 μM chlorpromazine, 150 μM dansylcadaverine or untreated (control) and incubated with TRITC-labelled transferrin. B. Vero cells transiently transfected with GFP-DIII∆2 or GFP-EH29 were incubated with TRITC-labelled transferrin. C. Cells were treated with 40 μM (A549) or 20 μM (HepG2 and U937) chlorpromazine and incubated with TRITC-labelled transferrin. (TIF) [file pone.0140824.s001.tif]

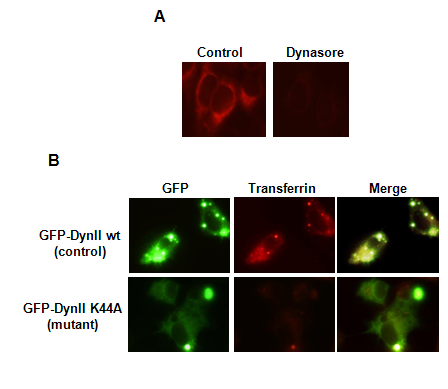

Supplement: S2 Fig — A. Vero cells were treated with 150 μM dynasore or untreated (control) and incubated with TRITC-labelled transferrin. B. Vero cells transiently transfected with GFP-Dyn II wt or GFP-Dyn II K44A were incubated with TRITC-labelled transferrin. (TIF) [file pone.0140824.s002.tif]

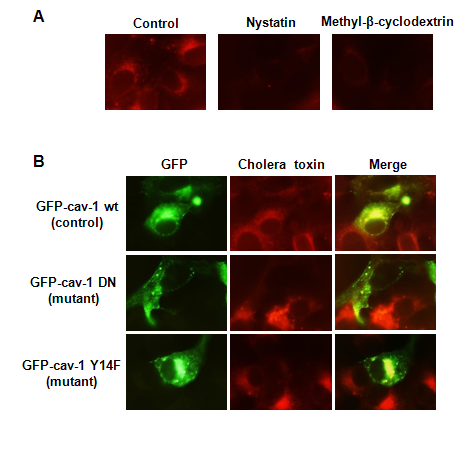

Supplement: S3 Fig — A. Vero cells were treated with 100 μM nystatin, 5 mM methyl-β-cyclodextrin or untreated (control) and incubated with TRITC-labelled cholera toxin subunit B. B. Vero cells transiently transfected with GFP-cav-1 wt, GFP-cav-1 DN or GFP-cav-1 Y14F were incubated with TRITC-Labelled cholera toxin subunit B. (TIF) [file pone.0140824.s003.tif]

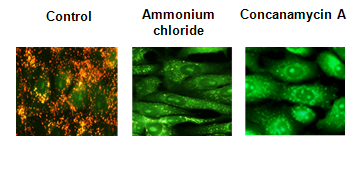

Supplement: S4 Fig — Vero cells were treated with 50 mM ammonium chloride, 10 nM concanamycin A or untreated (control) and stained with acridine orange. (TIF) [file pone.0140824.s004.tif]
